# Effects of Mitochondrial DNA Rate Variation on Reconstruction of Pleistocene Demographic History in a Social Avian Species, *Pomatostomus superciliosus*

**DOI:** 10.1371/journal.pone.0106267

**Published:** 2014-09-02

**Authors:** Janette A. Norman, Caroline J. Blackmore, Meaghan Rourke, Les Christidis

**Affiliations:** 1 National Marine Science Centre, Southern Cross University, Coffs Harbour, New South Wales, Australia; 2 Department of Genetics, University of Melbourne, Parkville, Victoria, Australia; 3 Museum Victoria, Melbourne, Victoria, Australia; 4 School of Science and Engineering, Deakin University, Geelong, Victoria, Australia; 5 New South Wales Department of Primary Industries, Narrandera Fisheries Centre, Narrandera, New South Wales, Australia; University of Sydney, Australia

## Abstract

Mitochondrial sequence data is often used to reconstruct the demographic history of Pleistocene populations in an effort to understand how species have responded to past climate change events. However, departures from neutral equilibrium conditions can confound evolutionary inference in species with structured populations or those that have experienced periods of population expansion or decline. Selection can affect patterns of mitochondrial DNA variation and variable mutation rates among mitochondrial genes can compromise inferences drawn from single markers. We investigated the contribution of these factors to patterns of mitochondrial variation and estimates of time to most recent common ancestor (T_MRCA)_ for two clades in a co-operatively breeding avian species, the white-browed babbler *Pomatostomus superciliosus*. Both the protein-coding ND3 gene and hypervariable domain I control region sequences showed departures from neutral expectations within the *superciliosus* clade, and a two-fold difference in T_MRCA_ estimates. Bayesian phylogenetic analysis provided evidence of departure from a strict clock model of molecular evolution in domain I, leading to an over-estimation of T_MRCA_ for the *superciliosus* clade at this marker. Our results suggest mitochondrial studies that attempt to reconstruct Pleistocene demographic histories should rigorously evaluate data for departures from neutral equilibrium expectations, including variation in evolutionary rates across multiple markers. Failure to do so can lead to serious errors in the estimation of evolutionary parameters and subsequent demographic inferences concerning the role of climate as a driver of evolutionary change. These effects may be especially pronounced in species with complex social structures occupying heterogeneous environments. We propose that environmentally driven differences in social structure may explain observed differences in evolutionary rate of domain I sequences, resulting from longer than expected retention times for matriarchal lineages in the *superciliosus* clade.

## Introduction

The use of mitochondrial DNA (mtDNA) as a marker for studying the demographic and evolutionary history of natural populations is widely established [1]. Several characteristics of the genome make it especially suited to this purpose including its relatively rapid rate of mutation (compared to single-locus nuclear genes), lack of recombination and ease of analysis. Of particular interest is the use of mtDNA to test for correlations between demographic and paleo-climatic events [2], an approach that has the potential to provide novel insights on how species might respond to future climate change.

Despite the widespread use of mtDNA variation to infer past demographic and evolutionary processes there are limitations to its use. Numerous studies have demonstrated that the mitochondrial genome shows departures from neutral equilibrium expectations due to the effect of selection (mutation-drift disequilibrium) or population processes (migration-drift disequilibrium). Failure to account for non-equilibrium conditions can lead to serious errors in the estimation of demographic parameters such as changes in effective population size and the timing of expansion or bottleneck events. Moreover, disequilibrium arising from different processes can produce similar genealogies, confounding the interpretation of violations from neutrality [3]. Selection for adaptive mutations may vary in the effect they have on populations of different sizes [4], leading to a weak or unpredictable relationship between mtDNA diversity and population size [5]. Population processes may lead to non-random mating and alter the relationship between genetic variation and demography. In species with sex-biased dispersal, for example, rare alleles can become fixed within philopatric social groups [6], increasing the amount of between-group variation leading to an effective population size greater than the number of breeding adults [7].

A second limitation derives from variation in mutation rate amongst individual mitochondrial genes or regions. Most studies to date rely on data from a single marker. Although individual mitochondrial genes are expected to reveal similar demographic histories due to their inheritance as a single linkage group, this assumption has not been widely tested. Nevertheless, comparisons of mutational patterns and evolutionary parameters derived from coding and non-coding portions of the mitochondrial genome have been informative. It has been shown that population growth rates can be significantly over-estimated when using mitochondrial markers that show departures from neutral equilibrium conditions [8]. High levels of site-specific rate heterogeneity in the control region can lead to reduced signals of divergence time relative to more slowly evolving protein-coding genes [9]. In a mitogenomic study, Duchêne et al [10] report considerable variation in age estimates derived from analysis of single gene regions and found that the control region, in addition to many slowly evolving protein coding regions, significantly over-estimated divergence times compared to complete mtDNA sequences. Other studies, however, have observed that similar evolutionary dynamics can be recovered by mitochondrial protein-coding genes and the control region, even though the information content of the regions may vary [11].

Variation in substitution rate across lineages can also lead to errors in the estimation of demographic parameters from mitochondrial sequence data. While it has become relatively commonplace to test the assumption of a strict molecular clock in higher-level phylogenetic studies, intra-specific phylogeographic data is usually modelled under the assumption of a strict molecular clock [12]–[15]. This is despite evidence that evolutionary rates can vary over time [16]-[19] and is time-dependent due to the presence of transient polymorphisms in recently evolved species or populations [20]–[22]. Alternatively, a relaxed clock model is applied but without explicitly testing the data for departures from a strict clock model [23], [24].

Here we use a range of statistics and coalescent approaches to evaluate the potential impact of departures from neutral equilibrium on Time to Most Recent Common Ancestor (T_MRCA_) estimates for two mitochondrial markers in the white-browed babbler *Pomatostomus superciliosus*. A sedentary, cooperative and sometimes plural breeder from southern Australia, it lives in social groups of up to 15 individuals [25] and defends breeding territories year-round [26]. Dispersal is female-biased, with males queuing to inherit the natal or neighbouring territory [27]. The presence of close relatives on the natal territory alters the potential for selection to occur [28] and may result in the over-representation of rare alleles within social groups [29]. The species range encompasses mesic to arid environments creating additional opportunities for local selection pressures to influence female reproductive fitness, patterns of gene flow and demographic history. Thus, the species provides an ideal model for investigating the potential effects of population- and marker-specific departures from neutral equilibrium conditions on estimates of T_MRCA_.

We analysed sequence variation in ND3, a protein-coding gene shown to detect macro-geographic genetic structure across a range of Australo-Papuan avian taxa [30]–[33], and in domain I of the control region. Although the control region is not universally variable among avian lineages [34], it shows rapid evolution and high variability in other Australo-Papuan babblers [35], [36]. Domain I is therefore likely to be sensitive to departures from equilibrium in white-browed babblers, and useful for testing the impact of departures from neutrality on estimates of divergence times.

## Materials and Methods

### Sampling

**Figure 1:**
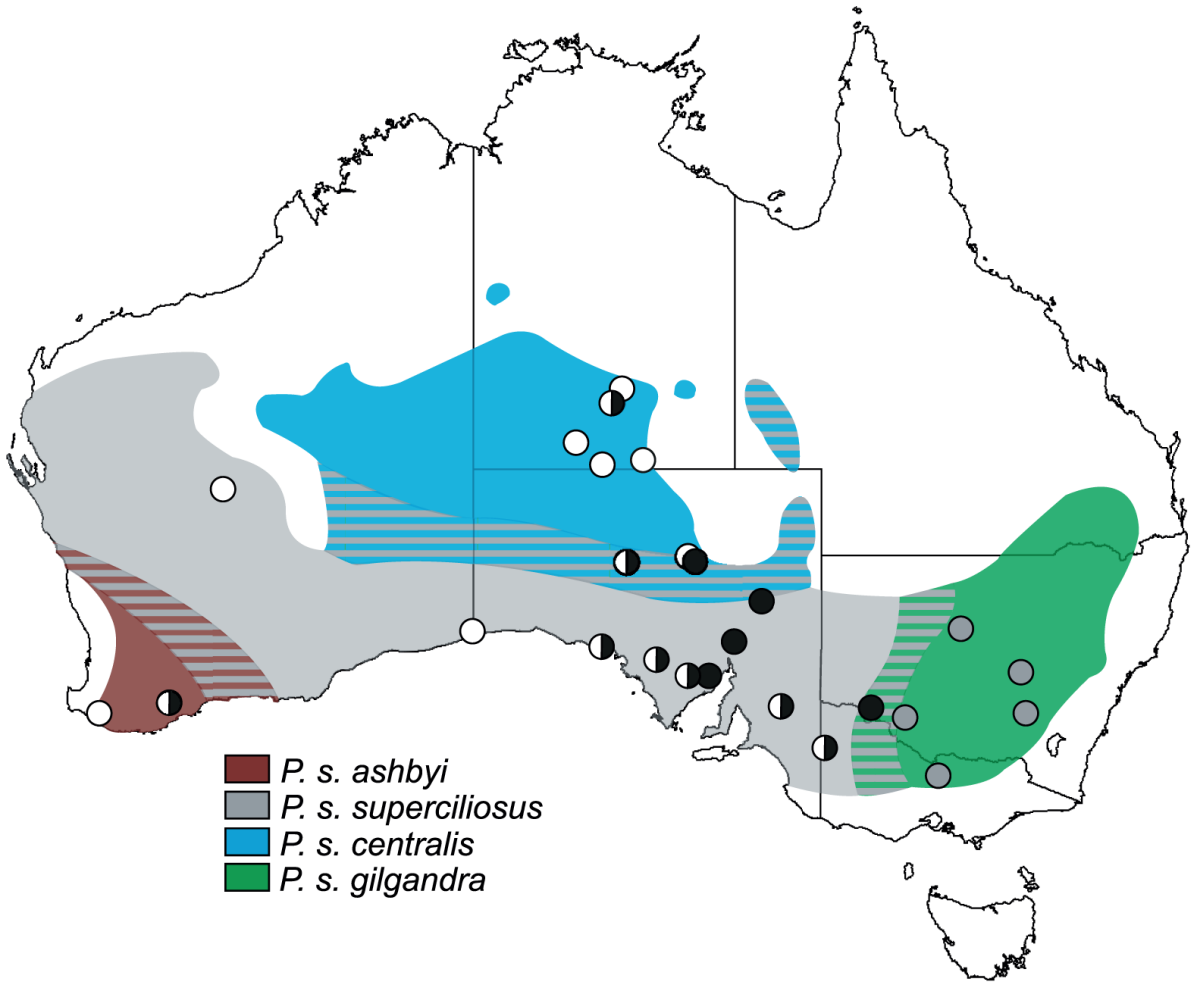
Map of Australia showing distribution of *P. superciliosus* subspecies and locations of sample sites. Cross-hatching denotes areas of possible intergradations between subspecies [39]. The occurrence of three domain I phylogroups (this study) are shown for each sampling location. Grey circles denote haplotypes unique to *P. s. gilgandra*. Black, white and bi-coloured circles denote, respectively, the presence of haplotypes from the eastern, western, and both eastern and western phylogroups within *P. s. superciliosus*. The one eastern haplotype (SCR-21) shown in the far south-west was found to cluster with the western phylogroup under alternative weighting schemes.

Tissue samples were sourced from researcher SV Edwards (Burke Museum) and the collections of Museum Victoria (Melbourne), Australian National Wildlife Collection (Canberra), Western Australian Museum (Perth) and Burke Museum of Natural History and Culture (Seattle, USA). These comprised 64 *P. superciliosus* from 26 sites across the species range in southern Australia (Figure 1, Table S1). Included were representatives of the four currently recognised subspecies [37]: *P. s. superciliosus* (n = 30), *P. s. centralis* (n = 10), *P. s. ashbyi* (n = 7) and *P. s. gilgandra* (n = 17). For outgroup comparisons and rate calibration we included single representatives from each of the following four taxa: Hall's babbler *P. halli*, the grey-crowned babbler *P. temporalis rubeculus*, the chestnut-crowned babbler *P. ruficeps*, and the rufous babbler *Garritornis isidorei* from New Guinea (formerly *P. isidorei*).

### Molecular analysis

Total cellular DNA was extracted from frozen or ethanol preserved liver, heart and muscle tissue using a salt:chloroform extraction method [38]. A 386 bp fragment of the mitochondrial genome containing the entire ND3 gene was amplified from all samples using the universal avian primers ND3-L10755 and ND3-H11151 [39]. A 413 base pair (bp) fragment from domain I of the mtDNA control region was amplified separately using the babbler-specific primers LD11 and HD14 [35]. PCR reactions were performed in 25 µl reaction volumes containing 0.1 µl *Taq* DNA polymerase (10 units/µl) and 2.5 µl 10X reaction buffer (Bresatec), 2.5 µl MgCl^2^ (30–60 mM), 0.5 µl of each dNTP (10 mM), 0.7 µl of forward and reverse primers (10 µM) and 12.5 µl of diluted DNA. Cycling conditions on a Hybaid Omn-E thermocycler were an initial 2 minute 95°C denaturing step, followed by 31 amplification cycles of 10 s denaturing at 94°C, 10 s of annealing at 55-58°C, and 60 s of extension at 72°C, with the extension step extended to 2–5 min in the final cycle. PCR products were visualised on an agarose gel to confirm amplification then purified using the UltraClean PCR Clean-up kit (MO BIO) prior to sequencing.

DNA sequencing was performed with gamma ^33^P-ATP end-labelled primers and the fmol DNA Sequencing System (Promega) according to manufacturer's instructions. Sequence ladders were read manually and the data aligned by eye. MEGA 4 [40] was used to check base composition, translate ND3 into amino acids and check for stop codons and indels. A BLAST search was conducted to ensure *P. superciliosus* ND3 and domain I sequences aligned with orthologous sequences from the avian mitochondrial genome, including published *Pomatostomus* control region sequences. ND3 sequences were truncated to 351 bp prior to subsequent analyses to remove short regions of flanking tRNAs. Sequences were submitted to GenBank under accessions KJ873913–KJ873962 (ND3) and KJ873963–KJ873961 (CR).

### Median-joining networks, summary statistics and neutrality tests

Relationships amongst ND3 and domain I haplotypes were reconstructed using median-joining network as implemented in the program Network 4.6.1.1 (Fluxus Technology Ltd). Networks for each marker were initially constructed using default weightings (10) for all sites. To minimise conflicts arising from homoplasies in the domain 1 network we applied a modified weighting scheme in which sites separating the major clades were up-weighted (20) and sites along terminal branches were down-weighted (5) leaving sites defining subclades of multiple haplotypes with the default weighting (10). Minor adjustments to the weighting scheme were applied to resolve, where possible, remaining ambiguities in the network.

Summary statistics of DNA polymorphism were calculated using DnaSP 5.10.01 [41] for each marker and each of the major clades (*superciliosus* and *gilgandra*) and subclades (eastern and western phylogroups) revealed from analysis of the median-joining networks. Statistics examined were number of haplotypes (*h*), haplotype diversity (*Hd*), number of segregating sites (*S*) and nucleotide diversity (π). Divergence between populations (*Dxy*) was also calculated. As population size expansion leads to changes in the frequency distribution of haplotypes (i.e., an excess of haplotypes or an excess of singleton mutations) we also calculated Strobeck's S statistic [42] and *R_2_* [43]. Strobeck's S statistic provides the probability of observing a sample that has the same or fewer haplotypes than *h* whereas *R_2_* contrasts the number of singletons and the mean number of differences amongst haplotypes. We also conducted tests of neutral equilibrium assumptions using two widely used statistics, Tajima's *D* [44] and Fu's *Fs* [45]. The 95% confidence intervals on point estimates of *h*, *R_2_*, *D* and *Fs* were obtained from 10,000 coalescent simulations conditioned on theta as implemented in DnaSP 5.10.1.

As both *D* and *Fs* have been shown to exhibit bias even under neutral conditions [46], [47], their interpretation, in the absence of other information, can be problematic. For mtDNA, *Fs* estimates the probability of observing a sample with the same or fewer haplotypes as the dataset given the observed value of diversity. A negative value indicates an excess of haplotypes, thus we should expect tests of departure from neutrality using *h* and *Fs* to be highly correlated. We also employed mismatch distributions as implemented in DnaSP 5.10.1 to determine if negative values of *D* or *Fs* were associated with haplotype distributions characteristic of a population expansion.

### Model selection and phylogenetic methods

Best fit models of sequence evolution were determined for each marker using the model selection procedures implemented in Kakusan 4 [48] with output files configured for analysis in MrBayes 3.2 [49]. As MrBayes 3.2 does not implement the full suite of models of sequence evolution evaluated by Kakusan 4 we also generated outputs for TreeFinder [50] which enabled a broader range of models to be evaluated. For the ND3 gene we determined best fit models for partitioned (by codon position) and unpartitioned data. Models were selected on the basis of AICc4 weightings [51] which use alignment length to assign the sample size for the data [48].

Phylogenetic relationships amongst haplotypes were inferred for each marker using MrBayes 3.2 with *P. temporalis* included as an outgroup based on a preliminary analysis of our data (not shown). This contrasts with a previous study of the mitochondrial cytochrome-*b* gene which identified *P. ruficeps* as the sister lineage to *P. superciliosus* [52]. The ND3 sequences were analysed using a codon partitioned model with rate matrices and gamma distributions unlinked across partitions. The K80, F81 and HKY85 models of substitution were specified for codon positions 1–3, respectively. For domain I, the best fit model of sequence evolution (TN93) was not implemented in MrBayes 3.2 so we used the HKY85 + Γ model. Bayesian MCMC simulations were run with four chains (1 cold, 3 hot) with 2 independent runs in each analysis and a coalescent tree prior. The first twenty-five percent of samples were discarded as burn-in and to ensure convergence of both runs we ran the MCMC in increments of 50,000–250,000 generations until the standard deviation of split frequencies was <0.01. Final MCMC runs comprised 750,000–5, 000,000 generations with trees drawn at intervals to provide a sample of 1,000 for the posterior distribution in each run. Potential scale reduction factors (PSRF) were evaluated at the completion of the run to ensure they were within the range 0.997<PSRF<1.003 indicating stationarity.

### Molecular clock testing

For both the ND3 and domain I datasets we evaluated whether the data conformed to an unconstrained, strict or relaxed clock model. Alternative models were evaluated from comparison of the marginal log likelihoods derived using the stepping-stone path sampling procedure implemented in MrBayes 3.2. Each analysis comprised 50 steps and 1,000,000 generations with the first sampling conducted close to the posterior distribution. For the unconstrained model we used the default branch lengths prior (brlenspr  =  unconstrained:exp[10.0]). For the strict and relaxed clock models we placed a uniform clock prior on branch lengths (brlenspr = clock:uniform) and used the clock rate variation prior to enforce a strict clock (clockvarpr = strict), or independent gamma rates (IGR) relaxed clock model (clockvarpr = IGR) with a default exponential prior distribution with rate 10.0. The IGR model estimates the rate at which the variance in effective branch length increases over time [53] and is the least complex of the relaxed clock models implemented in MrBayes 3.2. For both the strict and relaxed clock models we imposed topological constraints to ensure that both analyses employed the same rooting, as rate variation near the root of the tree can cause inconsistencies between analyses [54].

### Control region rate calibration and T_MRCA_ estimation

Two methods were used to determine an evolutionary rate for the domain I sequences. First, we used a simple regression analysis to compare divergence rates obtained for ND3 and domain I sequences from the representative outgroup taxa. We included single representatives from each of the major clades in *P. superciliosus* for comparison to determine whether the point estimate obtained using an ingroup comparison was higher than calculated for the outgroups as expected under a time-dependent model of rate variation. The ingroup sequences were chosen to represent the most common haplotypes present within each clade. A rate calibration of 0.0115 substitutions/site/lineage/MY (s/s/l/MY) was applied to the ND3 data as described below.

Secondly, we used BEAST 1.7.5 [55] to estimate a domain I clock rate. For this analysis we first used the ND3 dataset to estimate T_MRCA_ for the root node and each of the major clades within *P. superciliosus* as identified by our phylogenetic analysis. We applied a normally distributed clock rate prior of 0.0115 s/s/l/MYwith a standard deviation of 0.007. As average molecular rates are broadly conserved across most avian orders [56], our rate was derived from application of an ND2 calibration previously calculated for Australo-Papuan passerines [57] and comparative data on ND2 and ND3 divergences in the genus *Amytornis* [30]. The ND3 rate employed here is similar to the rate recently derived for this gene in Hawaiian honeycreepers [58]. We assume that the cited rate of 0.024 substitutions/site/MY is actually the divergence rate between lineages giving a per lineage substitution rate of 0.012 s/s/MY. To approximate the parameters of the best fit model of sequence evolution for the ND3 gene we employed the HKY85 model of sequence evolution. Analyses were run for 15,000,000 generations under a coalescent constant population size model with a Jeffrey's (1/x) prior and monophyly of the major clades enforced. Outputs were evaluated using Tracer 1.5 [59] to ensure ESS values were >200 after excluding the first 10% of samples as burn-in. The analysis was repeated three times and the results of the independent runs combined using LogCombiner v1.6.2 [60]. This provided T_MRCA_ estimates for the root node and each of the major clades.

To simultaneously derive a clock rate and T_MRCA_ estimates for the domain I sequences we used BEAST 1.7.5 to constrain the root age to the mean T_MRCA_ estimate obtained for this node in the previous analysis. We applied a strong normal prior to the root height with mean 1.43±0.0001 MY under the best fit TN93 model of sequence evolution. We ran three separate models to determine how our choice of priors affected the estimates. The initial model employed a coalescent constant population size tree prior and a strict molecular clock as described above. For the second model we allowed for rate variation by applying a random local clock which assumes a minimum number of rate changes. Unlike relaxed clock models that allow each branch to have a distinct rate, this model assumes rate changes subtend clades, thus allowing independent rates for the *gilgandra* and *superciliosus* clades. In the third model, T_MRCA_ and clock rate estimates were calculated using a coalescent population expansion tree prior. To determine appropriate values for the growth rate prior of the expansion model, we set the scale parameter to 1 and 10 in separate analyses and used Bayes Factor comparisons to evaluate the best fit model. As we did not find strong support for either model our final analysis used a Laplace prior for the growth rate with a scale factor of 3. Final MCMC runs comprised 10,000,000–50,000,000 generations (10,000 samples/run) with the longest runs being for the random local clock model. Finally, to further investigate the potential impacts of rate variation in the domain I sequences on T_MRCA_ estimates we constrained the age of the *gilgandra* and *superciliosus* clades to the mean T_MRCA_ estimates obtained for ND3 and determined the age of the root node and an alternative clock rate.

### Ethics statement

Ethics approval for this project was not required as all tissues samples were sourced from other researchers or specimens lodged in state museum collections as outlined in Table S1.

## Results

### Phylogeographic structure

**Figure 2:**
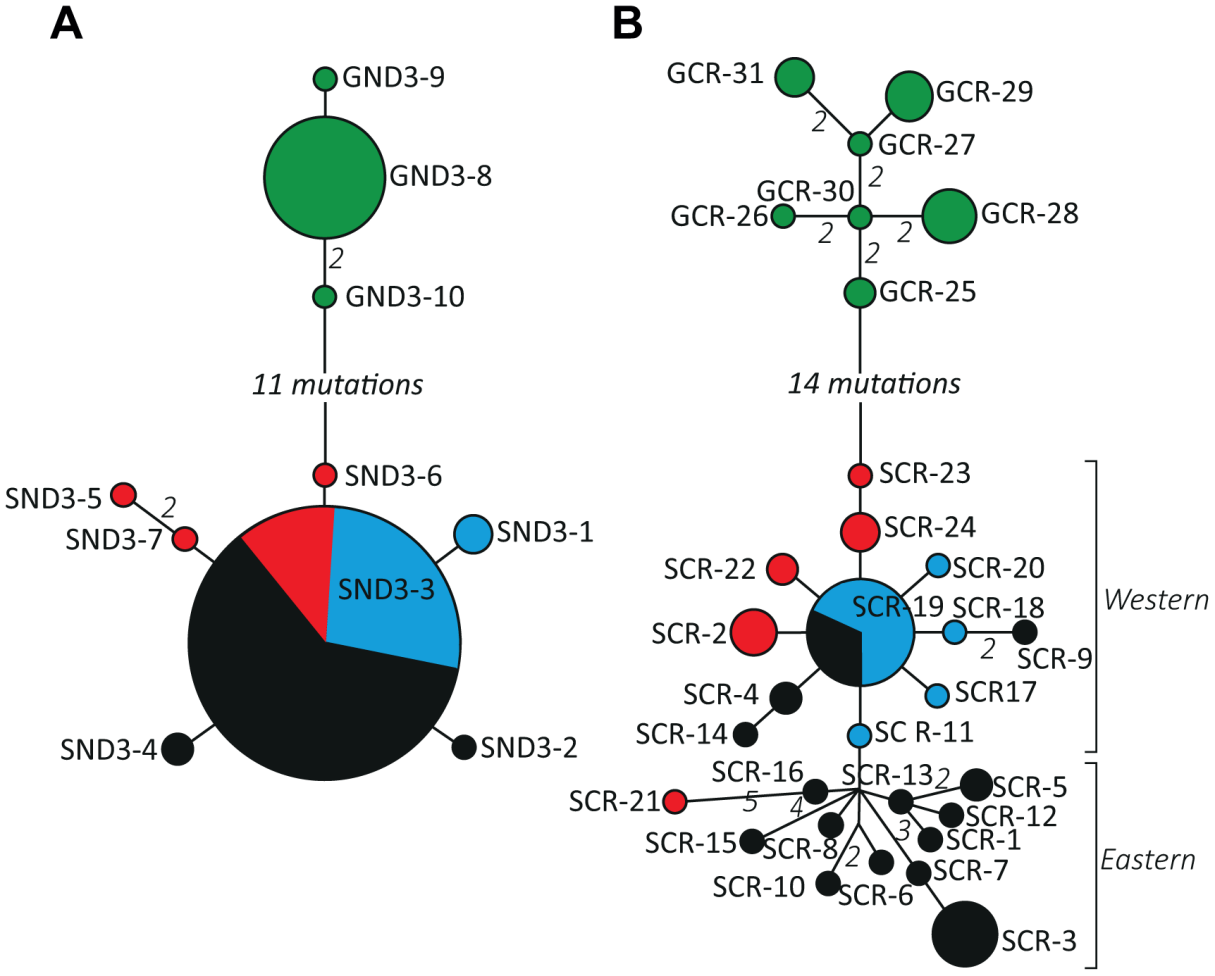
Median-joining haplotype networks for ND3 and domain I sequences. Circles indicate unique haplotypes with the area proportional to haplotype frequency. Green, black, red and blue circles represent the subspecies *P. s. gilgandra, P. s. superciliosus, P. s. centralis and P. s. ashbyi* as in Figure 1. Where a haplotype was sampled in more than one subspecies, pie slices are proportional to the number of individuals. Connections between haplotypes are single mutations unless indicated otherwise.

Visualisation of ND3 and domain I haplotype relationships using median-joining networks confirmed the presence of two major clades within *P. superciliosus*. One clade corresponded to *P. s. gilgandra* and the other comprised all samples assigned to the subspecies *P. s. superciliosus, P. s. ashbyi* and *P. s. centralis* (Figure 2), hereafter referred to as the *gilgandra* and *superciliosus* clades respectively. Divergence between clades (*Dxy*) was high, 3.24% for ND3 and 4.3% for domain I. Similar levels of population divergence in domain I sequences have been reported for Hall's babbler (3.29%) [36], and the grey-crowned babbler (4.53% in *P. t. temporalis* and 2.32% in *P. t. rubeculus*) [35].

The domain I network also revealed the presence of weak phylogeographic structure within the *superciliosus* clade but this did not correspond to recognised subspecies boundaries. Based on the geographic distribution of haplotypes we refer to the domain I subclades as the eastern and western phylogroups. The western phylogroup contains nearly all haplotypes sampled in the western and northern portions of the range of *superciliosus* and encompassed the forms previously designated as *P. s. ashbyi* and *P. s. centralis*. Additionally, some haplotypes sampled from the range of *P. s. superciliosus* were present. Haplotype SCR-21 sampled in the far south-west (*P. s. ashbyi*), although shown as a member of the eastern phylogroup (Figure 2), was found to cluster with the western phylogroup under alternative weighting schemes in our network analysis. Remaining haplotypes formed the eastern phylogroup and were restricted to the eastern limit of the subspecies' range.

### Polymorphism and neutrality tests

The ND3 and domain I sequences of *P. superciliosus* harboured variable levels of diversity (Table 1). Only 10 haplotypes were present among the 64 sampled ND3 sequences whereas 31 haplotypes were identified amongst the domain I sequences. Similarly, estimates of haplotype diversity (*H* 0.604, 0.949) and nucleotide diversity (π 0.0137, 0.0218) were consistently higher for domain I. ND3 sequences showed similar levels of haplotype and nucleotide diversity within the *gilgandra* and *superciliosus* clades. In contrast, domain I diversity was substantially higher in the *superciliosus* clade (*Hd* 3.4, π 0.0083) compared to *gilgandra* (*Hd* 2.2, π 0.0054). The eastern phylogroup (*Hd* 4.5, π 0.0109) also harboured more diversity than the western phylogroup (*Hd* 1.4, π 0.0063). The high levels of polymorphism in the domain I sequences of *P. superciliosus* are comparable to those previously reported for populations of the grey-crowned babbler (*P. temporalis rubeculus*) [35] and Hall's babbler [36].

**Table 1:** Summary statistics for DNA polymorphism and neutrality tests.

Partition/Population	*h*			*Hd*	*π*	*S*	*R2*		*D*		*Fs*	
ND3 (Total)	10	8–19	0.17	0.604	0.137	18	0.130	P = 0.828	0.757	P = 0.569	2.259	P = 0.948
gilgandra	3	1–5	0.87	0.324	0.0013	3	0.161	P = 0.402	−1.377	P = 0.053	−0.502	P = 0.312
superciliosus	7	1–6	**0.99**	0.346	0.0013	7	0.054	P = 0.058	**−1.903**	**P<0.001**	**−5.161**	**P<0.001**
CR (Total)	31	13–26	**1.00**	0.949	0.0218	39	0.111	P = 0.671	0.126	P = 0.631	−7.485	P = 0.03
gilgandra	7	2–9	0.91	0.853	0.0054	9	0.116	P = 0.197	−0.618	P = 0.297	−1.271	P = 0.215
superciliosus	24	5–15	**1.00**	0.924	0.0083	26	**0.059**	**P = 0.047**	−1.397	P = 0.064	**−15.259**	**P<0.001**
- eastern	13	5–12	**0.99**	0.905	0.0109	20	0.098	P = 0.172	−0.678	P = 0.284	−3.698	P = 0.045
- western	11	2–8	**1.00**	0.800	0.0043	13	**0.063**	**P<0.001**	**−1.607**	**P = 0.03**	**−7.351**	**P = 0.002**

CR, control region domain I. Number of haplotypes (*h*), expected range, and Strobeck's S statistic; Haplotype diversity (*Hd*); Nucleotide diversity (*π*); Number of segregating sites (*S*); Ramos-Onsins & Rozas goodness of fit statistic (*R_2_*) and probability calculated from coalescent simulations of null distribution; Tajima's D test of neutrality (*D*) and probability calculated from coalescent simulations of null distribution; Fu's test of neutrality (*Fs*) and probability calculated from coalescent simulations of null distribution. *Fs* is considered significant at P<0.02 [46]. Significant values in bold.

Significant departures from neutral expectations were observed for both markers (Table 1). The ND3 gene conformed to neutral expectations as measured by *h*, *R*_2_, *D* and *Fs* for the total sample and the *gilgandra* clade. However, departures from neutral equilibrium were observed for *superciliosus* as measured by *h*, *D* and *Fs*. A slight excess of haplotypes was observed for this clade but values of *S* and π were comparable to those observed in *gilgandra*. This suggests demographic factors or sampling error, rather than selection, is the cause of disequilibrium. For domain I, significant departures from neutral expectations were observed for the *superciliosus* clade and the western phylogroup for two or more statistics. For the *superciliosus* clade, a large excess of haplotypes (*h*) was observed (24 compared to an expected range of 5–15 predicted from null distributions). An excess of haplotypes was supported by a significantly low value for *R_2_* and a strongly negative *Fs*; signatures characteristic of haplotype distributions following expansion growth. Tajima's *D* was also negative but not statistically significant. Within *superciliosus*, the western phylogroup showed strong departures from neutral equilibrium expectations, consistent with population expansion, for *h* and *R_2_* and significantly negative values for *D* and *Fs*. Values for *S* and π were low compared to other clades suggesting a relatively recent period of population growth.

**Figure 3:**
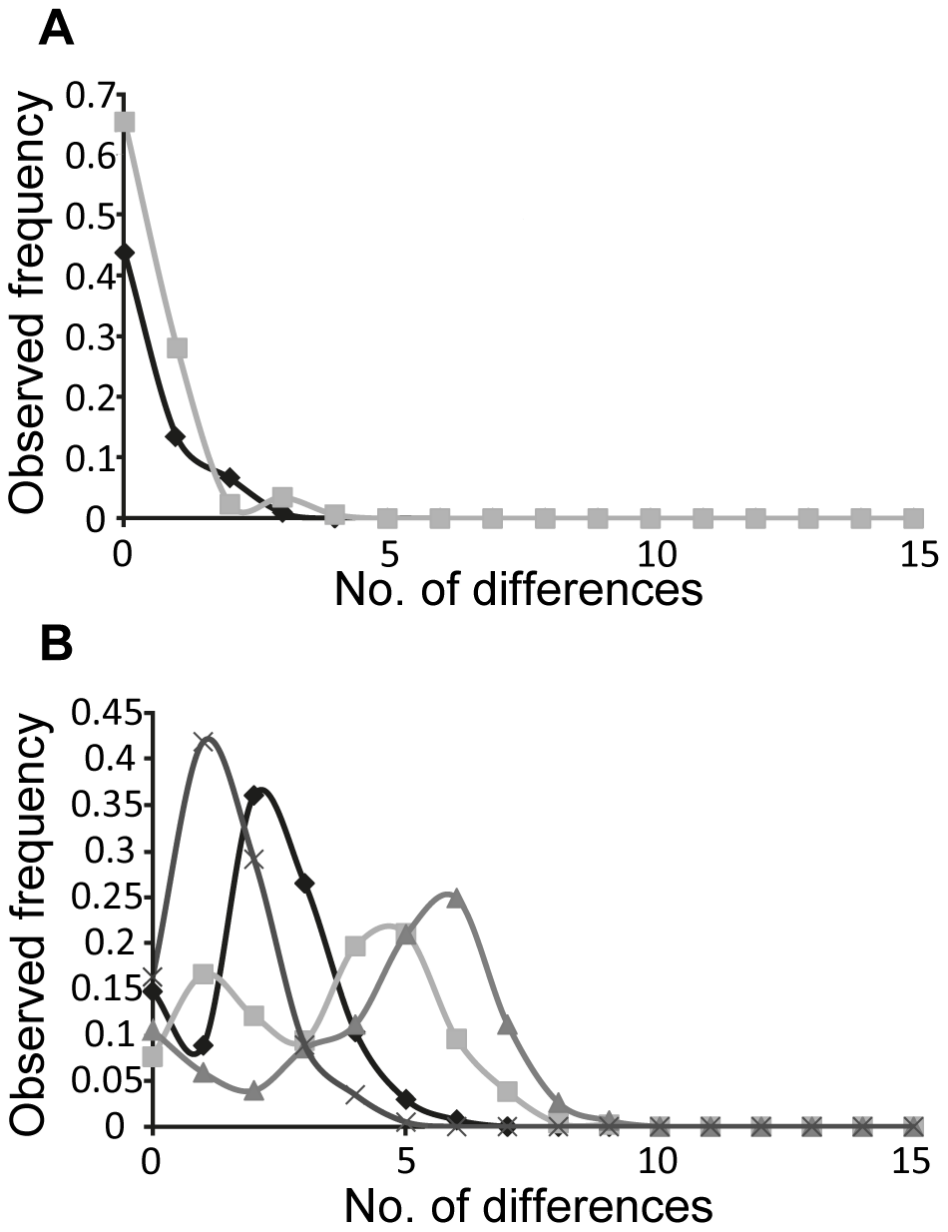
Mismatch distributions for *P. superciliosus* clades. (A) Mismatch distributions for *gilgandra* (diamonds) and *superciliosus* (squares) inferred using the ND3 sequences and a constant population size model. (B) Mismatch distributions for *gilgandra* (diamonds), *superciliosus* (squares), eastern phylogroup (triangles) and western phylogroup (crosses) inferred using domain I sequences and a constant population size model.

The ND3 and domain I sequences reveal contrasting demographic histories for the species when assessed using mismatch distributions (Figure 3). ND3 conformed to a constant population size model for both clades whereas as a growth/decline model was suggested for all clades when assessed using the domain I data. Multiple peaks in the mismatch distributions for the *superciliosus* clade were taken as evidence of population substructure. To test this, mismatch distributions were calculated separately for the eastern and western phylogroups. Despite all clades conforming to a population growth/decline model, the *R2* statistic (Table 1), a measure of goodness-of-fit, only provided support for expansion growth in the western phylogroup.

### Phylogenetic reconstruction and testing molecular clock models

**Figure 4:**
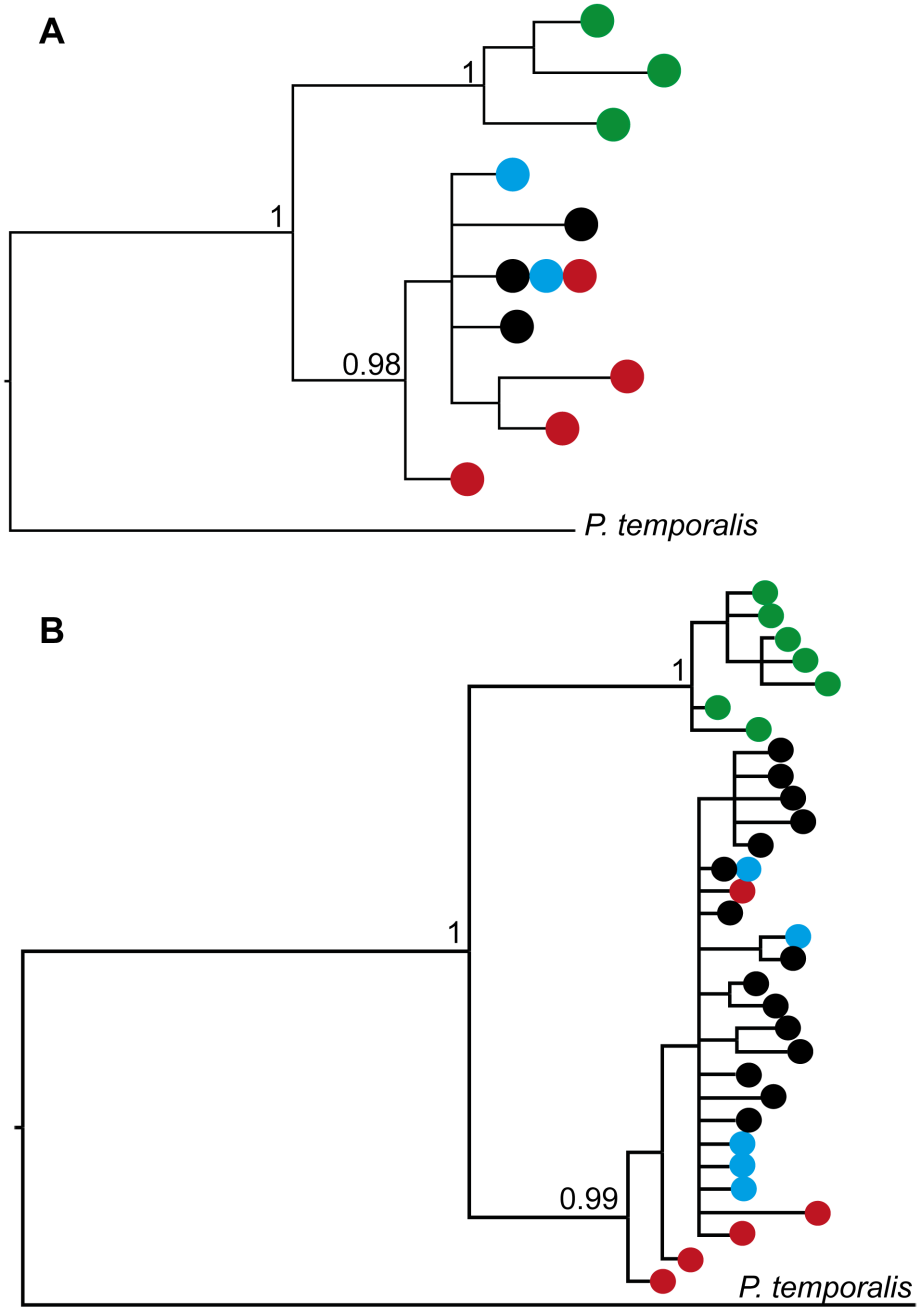
Clade structure for *P. superciliosus* inferred from Bayesian phylogenetic analysis. Bayesian consensus trees reconstructed from ND3 and domain I sequences showing posterior probabilities of the major nodes. Coloured circles at the branch tips denote the presence of that haplotype in each of the named subspecies; *P. s. gilgandra* (green), *P. s. superciliosus* (black), *P. s. centralis* (red) and *P. s. ashbyi* (blue). Colour-coded as in Figures 1 and 2.

Bayesian phylogenetic reconstructions of the ND3 and domain I sequences confirmed the presence of two major clades within *P. superciliosus* with high support for monophyly of the clades designated *gilgandra* and *superciliosus* (Figure 4). Sequences assigned to the forms *P. s. superciliosus*, *P. s. ashbyi* and *P. s. centralis* failed to resolve as reciprocally monophyletic groups, a finding consistent with haplotype relationships inferred using median-joining networks.

Marginal log likelihoods provided strong evidence against an unconstrained model for both markers. For the ND3 partition marginal log likelihoods failed to provide unequivocal support for a relaxed clock model (−726.02) over a strict clock model (−727.23) and we employed a strict clock model in subsequent analyses. For the domain I partition we found strong support for the IGR relaxed clock model (−1173.81) over the strict clock model (−1187.74), with 5 log units considered strong evidence in favour of the better model [61]. The posterior distribution of IGR branch rates revealed an increased evolutionary rate in the *superciliosus* clade with a strongly positive median value (12.6) separating that bipartition (IGR rates >1.0 indicate an increased rate when comparing bipartitions [54]). A positive value was also obtained for the bipartition separating *gilgandra* (8.4). We note that the 95% highest posterior density (HPD) interval for both clades is large indicating considerable uncertainty in rate estimation.

Bayesian coalescent analysis employing BEAST 1.7.5 did not support a departure from a strict clock model of sequence evolution in *P. superciliosus* domain I sequences. Bayes Factor comparisons of the harmonic mean estimator returned a smaller marginal log likelihood value (−972.42) for the random local clock model over a strict clock model (−973.08). However, this is a less robust method of model evaluation than stepping stone path sampling [62]–[64] and we cannot rule out that a rate change may have occurred during the evolution of the group, as indicated by our previous analysis.

### Control region domain I rate calibration

Regression analysis of pairwise values of percent sequence divergence in representative ingroup and outgroup samples was used to calculate a mean rate of 0.0143±SD s/s/l/MY for domain I. The point estimate obtained for the ingroup comparison [0.0148] was slightly higher than the mean rate, as predicted under a time-dependent model of evolutionary rates, but was similar to point estimates obtained in comparisons involving the outgroup taxon *G. isidorei*. This degree of variance is typical of mitochondrial rate calibrations; thus we take the mean rate as the best estimate of the evolutionary rate for the domain I sequences of *P. superciliosus* using this approach.

Our second approach to rate calibration involved constraining the tree root height (equivalent to the age of the root node) using the T_MRCA_ estimate obtained from Bayesian coalescent analysis of the ND3 gene (Table 2). This returned a mean clock rate of 0.02 s/s/l/MY, under a TN93 model, with a constant population size tree prior. Alternative models, in which we applied either a population expansion prior or a random local clock, produced similar estimates (Table 2). These rates are faster (∼1.4 times) than those calculated using a simple regression analysis based on limited ingroup and outgroup comparisons. Nevertheless, both methods are consistent in indicating that the non-coding domain I sequences evolve at a faster rate than those of the protein coding ND3 gene.

**Table 2:** Control region domain I clock rate calibrations and T_MRCA_ estimates.

	ND3	Control Region Domain I			
Parameters		Constant	Expansion	Local Clock	Internal
T_MRCA_ root node	1.429	-	-	-	0.853
					(0.519–1.248)
T_MRCA_ gilgandra	0.281	0.258	0.288	0.259	-
		(0.089–0.461)	(0.104–0.492)	(0.087–0.461)	
T_MRCA_ superciliosus	0.198	0.485	0.506	0.482	-
		(0.247–0.752)	(0.27–0.769)	(0.229–0.769)	
T_MRCA_ eastern	-	0.482	-	-	0.197
		(0.241–0.748)			(0.197–0.198)
T_MRCA_ western	-	0.448	-	-	0.191
		(0.188–0.73)			(0.143–0.198)
Clock rate	0.0115	0.020	0.018	0.020	0.0348
		(0.013–0.028)	(0.011–0.025)	(0.013–0.028)	(0.022–0.048)

Control region domain I clock rate calibrations (substitutions/site/lineage/MY) and mean T_MRCA_ estimates (MY) with 95% HPD intervals in brackets below. Rates estimated under a constant population size coalescent (Constant), expansion model (Expansion) and random local clock model (Local Clock) were constrained using the age of the root node calculated for ND3. Rates were also estimated by constraining the ages of *gilgandra* and *superciliosus* to the ND3 dates obtained for these nodes (Internal) and employing a constant population size coalescent.

Constraining the age of *gilgandra* and *superciliosus* to the T_MRCA_ estimates obtained for ND3 produced a substantially higher clock rate for the domain I sequences (mean 0.035 s/s/l/MY) and returned a substantially younger age for the root node (0.853 MY) (Table 2). This suggests an increase in evolutionary rate has occurred in association with the diversification of extant lineages within the *superciliosus* clade.

### Estimating T_MRCA_

From the ND3 gene we estimated T_MRCA_ for the root node and the two major clades, *gilgandra* and *superciliosus* (Table 2). Our T_MRCA_ estimate for the root node of 1.43 MY suggests that these clades diverged during the mid-Pleistocene, with the T_MRCA_ estimates for *gilgandra* (0.281 MY) and *superciliosus* (0.198 MY) indicating late-Pleistocene diversification of lineages within both clades. Although a more recent origin for sequences of the *superciliosus* clade is indicated, the 95% HPD intervals on both estimates broadly overlapped.

Under a model of constant molecular evolution and neutral equilibrium conditions (constant population size and panmixia) we would expect domain I sequences to return similar T_MRCA_ estimates using the clock rate calibrated in this study. Consistent with neutral equilibrium expectations domain I sequences produced similar T_MRCA_ estimates for *gilgandra* to those estimated for ND3, irrespective of the model considered, with means ranging from 0.258 to 0.288 MY (Table 2). In contrast, domain I T_MRCA_ estimates for the *superciliosus* clade (0.485 to 0.506 MY across the three models) were substantially higher than the mean ND3 estimate. T_MRCA_ estimates for the eastern and western phylogroups were also high at 0.482 and 0.448 MY, respectively. Given the evidence of strong departures from neutral equilibrium conditions and variation in rates of molecular evolution, we contend that domain I T_MRCA_ values for *superciliosus*, and the eastern and western phylogroups, are substantially over-estimated.

## Discussion

### Neutral equilibrium and variation in evolutionary rate

We detected significant departures from a neutral model of molecular evolution in the mtDNA of *P*. *superciliosus*. All tests showed disequilibrium in the western phylogroup resulting from an excess of domain I haplotypes, a mutational pattern consistent with recent population expansion in this part of the species' range. However, the tests varied considerably in their ability to detect departures from neutrality in other population groupings. Furthermore, standard neutrality tests were not appropriate for detecting disequilibrium conditions resulting from observed variation in evolutionary rate of domain I sequences. Together, these findings demonstrate a strong time-dependent effect of molecular evolution in *P. superciliosus* driven, in part, by spatially heterogeneous demographic processes.

The application of neutrality tests to detect departures from mutation-drift or migration-drift equilibrium can be problematic. The wide array of available statistics is based on different assumptions (e.g. finite versus infinite alleles models), utilise different parameters, and display different sensitivities and biases even under neutral conditions. Consequently, neutrality tests typically do not provide consistent support for neutral evolution making the choice of tests critical. Tajima's *D* tends to show a positive bias even under neutral conditions and is relatively insensitive to changes in frequency spectrum due to small increases in haplotype number and singleton mutations. We used it to provide a conservative comparison against two statistics that are reported to be more suitable for detecting population expansion: *Fs* and *R_2_* [44], [65]. *Fs* has been shown to be better for large samples and *R_2_* for small samples [44]; thus we expected these statistics would perform differently based on the size of the populations under study.

The power of neutrality tests is a function of sample size (n) and the number of segregating sites (S). Simulations have shown that *R_2_* is the most powerful where both parameters are small, with a power of 0.5 when n∼10 and S∼5 [44]. Of the eight *P. superciliosus* haplotype groupings assessed only one did not exceed these parameters (*gilgandra* ND3, n = 17, S = 3), and our ability to detect non-neutral signatures may be low for this sample. As sample size increases, *Fs* becomes the more powerful test with a power of 0.5 when n∼50 and S∼7. This vulnerability to changes in n and S argues for the use of multiple tests to maximise power across the range of sample sizes and patterns of diversity that natural populations are likely to present. Choice of statistics cannot be determined *a priori* as the actual structure and size of populations, and the number of segregating sites, is unknown. Sampling strategies based on taxonomic groups are often incorrect, so alternative classifications must be identified and applied to the data before analysis, as in the present study.

We found *Fs* to be the most consistent predictor of deviations from neutrality for our dataset. For both ND3 and the domain I sequences, the number of tests providing support for non-neutral evolution increased as the hierarchical level decreased, from analysis of the total sample to phylogroups. For domain I sequences the total data showed a significant departure from expectations for *h* only. When comparing the major clades *R_2_* and *Fs* returned significant results for *superciliosus* (but not *gilgandra*), while all three neutrality tests showed evidence of non-neutral evolution for the western phylogroup. Thus, the observation of non-neutral evolution at higher levels appears to be driven by large deviations from neutrality in the western phylogroup. Our analysis also provides empirical support for the findings of Ramos-Onsins and Rozas [44] that *Fs* is a sensitive indicator of departures from neutrality under population expansion, despite simulations that suggest this statistic is relatively insensitive to such perturbations [65].

Our analysis also provides evidence for variation in the evolutionary rate of domain I sequences. Estimation of the clock rate using Bayesian coalescent simulations suggests an almost two-fold increase in evolutionary rate associated with diversification of the *superciliosus* clade. Despite an increased level of divergence amongst *superciliosus* haplotypes this did not result in departures from neutrality as estimated by Tajima's *D* as both the number of segregating sites and nucleotide diversity increased proportionally (Table 2). Tests of molecular clock assumptions are not routinely included in phylogeographic analyses but we show here that variation in evolutionary rate can be an important source of deviation from neutral expectations that standard statistics, such as Tajima's *D* and *Fs*, may not account for.

An important consequence of the observed variation in evolutionary rate within the domain I sequences is that subsequent estimates of T_MRCA_, and inferences of demographic history derived from them, are similarly distorted. Given that our analyses indicate that the ND3 gene is evolving at an approximately constant rate, and no departures from neutrality were evident for *gilgandra* or the total data set, we consider the T_MRCA_ estimates derived from this dataset to be the most reliable for reconstructing demographic events. Mean ND3 T_MRCA_ estimates for *gilgandra*, *superciliosus* and the divergence of these clades (the root node) were 281,000 years, 198,000 years and 1.43 million years, respectively. While the domain I sequences returned a similar mean estimate and 95% HPDs for *gilgandra* (258,000 years under a constant population size model) values for *superciliosus* were considerably over-estimated at 485,000 years, a more than two-fold increase. There was also minimal overlap in the 95% HPDs of these two estimates (0.07–0.35 MY and 0.25–0.75 MY, respectively). By constraining the age of the *gilgandra* and *superciliosus* clades to the ND3 determined ages we also demonstrate that variation in evolutionary rate causes the domain I sequences to substantially under-estimate the age of the root node, placing this at 853,000 years rather than 1.43 MY. Furthermore, the entire 95% HPD of the domain I estimate was below the mean ND3 estimate. Thus, depending on the choice of node constraints (root node or internal nodes), rate variation in the domain I sequences can cause T_MRCA_ to be substantially over- or under-estimated.

Considerable variation in T_MRCA_ estimates can also arise through the choice of rate calibration methods. Simple correlative approaches based on limited comparisons returned substantially lower estimates of evolutionary rate than coalescent based analyses of the complete dataset (0.014 cf. 0.02). Using the former estimate would return considerably older estimates of T_MRCA_ for *P. superciliosus*. While the current trend towards mitogenomic sequencing will eventually yield larger datasets and the potential to reduce some sources of error in evolutionary estimation [10], the impact of marker-specific rate variation and calibration methods on the resulting estimates will need to be incorporated.

### Pleistocene diversification

The demonstration of a greater than two-fold difference in estimates of T_MRCA_ for some evolutionary events has important implications for the use of mitochondrial variation for reconstructing demographic history, especially inferences concerning the impact of Pleistocene climate events on species evolution. The Pleistocene climatic history of Australia is dominated by consecutive phases of increasing aridification [66], [67], as well as a marked change in the seasonality of rainfall across the south-east of the continent at approximately 1.5 MYA [68]. Southern hemisphere glacial activity did not commence until the mid Pleistocene (∼750,000 years ago) and intensified during the mid-Brunhes transition ∼420,000 years ago [69]. Only three glacial incursions are recorded for the southernmost parts of the continent [70], at marine isotope stage (MIS) 10 (360,000 years), MIS 6 (130,000–190,000 years) and MIS 2 (14,000–29,000 years) [71], with glaciations impacting the mainland only during the last glacial maximum [72].

Our ND3 estimates of T_MRCA_ suggest that aridification and changes in the seasonality of rainfall during the early Pleistocene may have been the main drivers of divergence between *gilgandra* and *superciliosus*. Alternatively, using the domain I T_MRCA_ of 853,000 years, obtained using internal node age constraints, we would more likely infer that it was significant cooling associated with the onset of southern hemisphere glaciations that promoted the divergence of these populations. Similarly, estimates from the ND3 gene suggest closely timed Pleistocene events (198,000–281,000 years) are associated with the onset of diversification of lineages within both clades whereas domain I sequences suggest that they are more likely to have been driven by temporally disparate events at 258,000 (MIS 7) and 485,000 years (MIS 12). We consider estimates derived from the ND3 dataset to be more reliable for making evolutionary inferences of Pleistocene population history for this species given demonstrated rate variation in the domain I sequences.

### Population structure and demography

The white-browed babbler, like its congeners, is a co-operatively breeding species that forms social groups and maintains discrete territories [26], [27]. Cooperative breeding involves the presence of non-breeding helpers that delay dispersal and are often the offspring of the resident breeding pair. Despite theoretical predictions that such behaviours would lead to high levels of population structuring and limited migration [29], mitochondrial studies of the grey-crowned and Hall's babblers have revealed evidence of long-distance gene flow and asymmetric migration between genetically divergent populations [35], [36]. Our study lacks the detailed population-level sampling required to assess inter-population migration but we find no evidence of female-mediated gene flow between *gilgandra* and *superciliosus*. The level of mtDNA divergence between these clades, and presence of reciprocal monophyly, supports their recognition at the subspecific level. We find no support for the long-term isolation of *P. s. ashbyi* and *P. s. centralis* in putative Pleistocene refugia. Instead, these populations form part of a large phylogeographic assemblage distributed across southern Australia and characterised by a more complex, low-level of phylogeographic structure.

We interpret the phylogeographic pattern in the *superciliosus* clade as evidence of a formerly widespread ancestral population characterised by high diversity in the eastern part of the range. Overlain on this is evidence of a recent population expansion in the western and north-central part of the range with extant haplotypes assorting into two closely-related phylogroups separated by a single mutational difference on domain I sequences. Haplotypes in the western phylogroup form a starlike phylogeny arising from the widespread ancestral haplotype, SCR-19, which occurred in populations from across the geographic range of *superciliosus*. The restricted distribution of haplotypes in the western phylogroup indicates some restriction on female-mediated gene flow amongst regional populations. Our sample sizes are, however, insufficient to determine if other factors have influenced the contemporary distribution of haplotypes. Areas of contact between eastern and western phylogroups may have arisen from incomplete lineage sorting, restricted gene flow, or a range expansion associated with population growth in the western and/or central-northern populations.

Significant differences in levels and patterns of mtDNA diversity were observed between *gilgandra* and *superciliosus* (Table 1). Most notably, *superciliosus* is characterised by the retention of a large number of moderately divergent and geographically widespread haplotypes, a pattern not evident in *gilgandra*. We postulate that this pattern has arisen as a result of differences in social structuring associated with cooperative breeding that has facilitated the persistence of maternal lineages, and longer than expected retention times for local polymorphisms [6], [29] in *superciliosus*. Consequently, temporal changes in social structure may be an important determinant of time-dependent molecular evolution and the shift in evolutionary rate observed in *P. superciliosus*.

Environmental heterogeneity may facilitate the evolution of variable social organisation and observed spatio-temporal patterns of mtDNA variation. Breeding success is strongly associated with group size in chestnut-crowned babblers [73] and the number of male helpers in grey-crowned babblers [74], with greater helper retention likely to be associated with aridity in grey-crowned babblers [75]. Resource availability also appears to influence juvenile dispersal in chestnut-crowned babblers [76] and habitat configuration can influence the size and arrangement of breeding territories [77], [78]. Together these studies suggest complex interactions between environmental heterogeneity, social structure and dispersal that not only influence reproductive success in babblers, but lead to considerable variance in the degree of spatial and temporal connectivity between social groups. We hypothesise that these factors may operate together to produce the geographically variable patterns of mtDNA population structure evident in *P. superciliosus* and related species.

Consistent with this hypothesis, strongly divergent social systems have evolved within *P. superciliosus*. Although described as a cooperative breeder with social groups consisting of a single breeding pair and auxiliary helpers [25], [27], [78], only *superciliosus* from the far south-west of the species range are single-pair cooperative breeders. In contrast, only plural breeding, with two to four breeding pairs per group, has been found in *P. s. gilgandra* [79], [80]. While the social organisation of the *superciliosus* clade across its range is unknown, the pivotal role of harsh ecological conditions [81], [82], [83], especially temporal variability in rainfall [84], on the evolution of cooperative breeding in birds, single-pair cooperative breeding could be expected throughout it's mostly arid- and semi-arid range.

This raises the possibility that spatio-temporal shifts in social organisation have occurred in *P*. *superciliosus* as an adaptive response to increasing Pleistocene aridification, with profound effects on population genetic structure. Strong local genetic structure arising from single-pair co-operative breeding [85], [86] could facilitate the retention of ancestral polymorphisms leading to the observed shift in evolutionary rate in this clade. Conversely, the dilution of matrilines through plural breeding [28] would bring *P. s. gilgandra* close to panmixia and maintain neutral equilibrium conditions. The potential impacts of spatio-temporal variation in social organisation on population genetic structure may be more widespread than currently recognised, especially in Australia where there is a high incidence of co-operative breeding in birds [87]. Integration of ecological and molecular studies will be required to disentangle these effects and is likely to be a productive area of research for investigating potential climate change adaptation responses in birds. Finally, spatio-temporal variation in social organisation may be an unrecognised factor contributing to the time-dependency of molecular evolution in recently evolved lineages.

## Supporting Information

Table S1 **Sample identification codes and source, collection locales, GenBank accessions, haplotype designations and specimen vouchers.** Institutional abbreviations are: MV, Museum Victoria; ANWC, Australian National Wildlife Collection; SAM, South Australian Museum; AM, Australian Museum; UWBM, University of Washington Burke Museum. SVE denotes tissue samples obtained from researcher Scott V. Edwards; all other samples were obtained from the source institutions indicated. Abbreviations used for collection locales are: VIC, Victoria; NSW, New South Wales; SA, South Australia; NT, Northern Territory; WA, Western Australia; QLD, Queensland; PNG, Papua New Guinea. Haplotype designations are the same as in Figure 2. Specimen voucher information is provided for all currently registered specimens in museum collections.
(DOC)
