# Supplementary material for: Effects of Mitochondrial DNA Rate Variation on Reconstruction of Pleistocene Demographic History in a Social Avian Species, Pomatostomus superciliosus
Source: PLoS One. 2014 Sep 2;9(9):e106267. doi: 10.1371/journal.pone.0106267 (PMC4152169; doi:10.1371/journal.pone.0106267)
Supplement: Table S1 — Sample identification codes and source, collection locales, GenBank accessions, haplotype designations and specimen vouchers. Institutional abbreviations are: MV, Museum Victoria; ANWC, Australian National Wildlife Collection; SAM, South Australian Museum; AM, Australian Museum; UWBM, University of Washington Burke Museum. SVE denotes tissue samples obtained from researcher Scott V. Edwards; all other samples were obtained from the source institutions indicated. Abbreviations used for collection locales are: VIC, Victoria; NSW, New South Wales; SA, South Australia; NT, Northern Territory; WA, Western Australia; QLD, Queensland; PNG, Papua New Guinea. Haplotype designations are the same as in Figure 2. Specimen voucher information is provided for all currently registered specimens in museum collections. (DOC) [file pone.0106267.s001.doc]

Table S1. Sample identification codes and source, collection locales, GenBank accessions, haplotype designations and specimen vouchers.

| Taxon | Sample ID | Source | Collection locale | ND3 GenBank accessions | CR GenBank accessions | Haplotypes | Specimen voucher |
| --- | --- | --- | --- | --- | --- | --- | --- |
| *P. s. gilgandra* | MV2767  MV2787  MV2788 | MV  MV  MV | Rushworth State Forest, VIC | KJ873921  KJ873923 | KJ873957  KJ873954  KJ873953 | GND3-8, GCR-30  GND3-8, GCR-31  GND3-10, GCR-28 | MV:B21578  MV:B21579  MV:B23609 |
|  | MV687  MV688 | MV  MV | Inglewood, VIC |  |  | GND3-8, GCR-28  GND3-8, GCR-28 | MV:B25390  MV:B20848 |
|  | B344  B345  B346 | MV  MV  MV | Jindalee State Forest, NSW |  | KJ873952  KJ873951  KJ873956 | GND3-8, GCR-25  GND3-8, GCR-26  GND3-8, GCR-27 | ANWC:B38266  ANWC:B24232 |
|  | LC184 | MV | Cobar, NSW |  |  | GND3-8, GCR-25 | ANWC:B38433 |
|  | FG25-25  FG26-30  FG27-33  FG28-35  FG29-41  FG30-50  FG31-56  FG32-44 | SVE  SVE  SVE  SVE  SVE  SVE  SVE  SVE | Blowclear West State Forest, NSW | KJ873922 | KJ873955 | GND3-8, GCR-29  GND3-8, GCR-29  GND3-9, GCR-28  GND3-8, GCR-31  GND3-8, GCR-29  GND3-8, GCR-29  GND3-8, GCR-31  GND3-9, GCR-28 | AM:O59319  AM:O59320  AM:O59321  AM:O60904  AM:O62716  AM:O65058  AM:O67641 |
| *P. s. superciliosus* | MV2794  MV2795  MV2809 | MV  MV  MV | Big Desert State Forest, VIC | KJ873917 | KJ873939 | SND3-3, SCR-19  SND3-3, SCR-19  SND3-4, SCR-12 | MV:B21581  MV:B21582  MV:B23608 |
|  | CES42 | UWBM | Mildura, VIC | KJ873913 | KJ873927 | SND3-3, SCR-1 | UWBM:57438 |

Table S1. Continued.

|  | W09  W035 | MV  MV | Mabel Creek, SA |  | KJ873931  KJ873932 | SND3-3, SCR-3  SND3-3, SCR-4 | SAM:B39204  SAM:B39203 |
| --- | --- | --- | --- | --- | --- | --- | --- |
|  | MV1361 | MV | Sinclair’s Gap, SA |  |  | SND3-3, SCR-3 | MV:B20014 |
|  | FG36-137  FG36-138  FG37-143  FG37-144  FG38-147  FG38-148 | SVE  SVE  SVE  SVE  SVE  SVE | Kimba, SA |  | KJ873934  KJ873937  KJ873935  KJ873938  KJ873936 | SND3-3, SCR-6  SND3-3, SCR-9  SND3-3, SCR-7  SND3-3, SCR-10  SND3-3, SCR-3  SND3-3, SCR-8 | SAM:B45207  SAM:B45208  SAM:B45213  SAM:B45214  SAM:B45217  SAM:B45218 |
|  | 28219  28220 | ANWC  ANWC | Port Augusta, SA |  |  | SND3-3, SCR-3  SND3-3, SCR-3 | ANWC:B28219 ANWC:B28220 |
|  | 28272 28273 | ANWC  ANWC | Ceduna, SA | KJ873915 | KJ873940 | SND3-2, SCR-13  SND3-3, SCR-4 | ANWC:B28272 ANWC:B28273 |
|  | 48246 48247 | ANWC  ANWC | Flinders Ranges, SA |  | KJ873942  KJ873943 | SND3-3, SCR-15  SND3-3, SCR-16 | ANWC:B48246  ANWC:B48247 |
|  | 46752 46753 | ANWC  ANWC | Eyre Peninsula, SA |  | KJ873941 | SND3-3, SCR-3  SND3-3, SCR-14 | ANWC:B46752  ANWC:B46753 |
|  | FG35-113 FG35-114 | SVE  SVE | Blanchetown, SA |  | KJ873933 | SND3-4, SCR-5  SND3-3, SCR-5 | SAM:B4516 |
|  | MV132 MV133 | MV  MV | Eucla, WA |  |  | SND3-3, SCR-19  SND3-3, SCR-19 | MV:B18022  MV:B18021 |
|  | CMH65 CMH66 PLG283 SVE1266 | UWBM  UWBM  UWBM  UWBM | Wiluna, WA |  | KJ873929 | SND3-3, SCR-2 SND3-3, SCR-2 SND3-3, SCR-2 SND3-3, SCR-2 | UWBM:60757  UWBM:60758  UWBM:60845  UWBM:60939 |
| *P. s. centralis* | D227 D228 | MV  MV | William Creek, SA |  | KJ873930 | SND3-1, SCR-19 SND3-1, SCR-11 | ANWC:B40203  ANWC:B40204 |
|  | 48962 48963 | ANWC  ANWC | Tieyon Head Station, SA |  | KJ873946 | SND3-3, SCR-19 SND3-3, SCR-20 | ANWC:B48962  ANWC:B48963 |

Table S1. Continued.

|  | D240 | MV | New Crown Station, NT |  |  | SND3-3, SCR-19 | ANWC:B40216 |
| --- | --- | --- | --- | --- | --- | --- | --- |
|  | MV1109 MV1110 | MV  MV | Stockyard Homestead, NT | KJ873916 | KJ873945  KJ873944 | SND3-1, SCR-18, SND3-3, SCR-17 | MV:Z333  MV:B20013 |
|  | 48831 | ANWC | Alice Springs, NT |  | KJ873928 | SND3-3, SCR-19 | ANWC:B48831 |
|  | 48911 48912 | ANWC  ANWC | MacDonnell Range, NT |  |  | SND3-3, SCR-19  SND3-3, SCR-19 | ANWC:B48911  ANWC:B48912 |
|  | 48943 | ANWC | Kulgera, NT |  |  | SND3-3, SCR-19 | ANWC:B48943 |
| *P. s. ashbyi* | 31888 31889 31890 31891 | ANWC  ANWC  ANWC  ANWC | Brockman State Forest, WA | KJ873918  KJ873920 | KJ873949  KJ873950 | SND3-6, SCR-23  SND3-3, SCR-24 SND3-7, SCR-24 SND3-3, SCR-24 | ANWC:B31888  ANWC:B31889  ANWC:B31890  ANWC:B31891 |
|  | 31738 31739 31740 | ANWC  ANWC  ANWC | Ongerup, WA | KJ873918 | KJ873947  KJ873948 | SND3-3, SCR-22 SND3-5, SCR-21  SND3-3, SCR-22 | ANWC:B31738  ANWC:B31739  ANWC:B31740 |
| *P. temporalis* | MV2561 | MV | Tom Price, WA | KJ873925 | KJ873959 |  | MV:B24209 |
| *P. halli* | JCW115 | MV | Winton, QLD | KJ873924 | KJ873958 |  | ANWC:B40002 |
| *P. ruficeps* | MV1020 | MV | Frome Downs Homestead, SA | KJ873926 | KJ873960 |  | MV:B25021 |
| *G. isidorei* | E692 | MV | Veimauri River, Central Province, PNG | KJ873914 | KJ873961 |  | MV:B19279 |

Table S1. Continued.

Institutional abbreviations are: MV, Museum Victoria; ANWC, Australian National Wildlife Collection; SAM, South Australian Museum; AM, Australian Museum; UWBM, University of Washington Burke Museum. SVE denotes tissue samples obtained from researcher Scott V. Edwards; all other samples were obtained from the source institutions indicated. Abbreviations used for collection locales are: VIC, Victoria; NSW, New South Wales; SA, South Australia; NT, Northern Territory; WA, Western Australia; QLD, Queensland; PNG, Papua New Guinea. Haplotype designations are the same as in Figure 2. Specimen voucher information is provided for all currently registered specimens in museum collections.
